# NSAIDs-Related Pyloroduodenal Obstruction and Its Endoscopic Management

**DOI:** 10.1155/2011/967957

**Published:** 2011-06-06

**Authors:** Mohd Talha Noor, Pankaj Dixit, Rakesh Kochhar, Birinder Nagi, Usha Dutta, Kartar Singh, Kuchhangi Suresh Poornachandra

**Affiliations:** Department of Gastroenterology, Postgraduate Institute of Medical Education and Research, Sector 12, Chandigarh 160012, India

## Abstract

Endoscopic balloon dilatation (EBD) has important role in the management of benign gastric outlet obstruction. Although there are many reports on the role of EBD in the management of corrosive-induced and peptic benign GOO, there is scanty data on its role in the management of NSAID-induced GOO. We report 10 cases of NSAID-induced pyloroduodenal obstruction and their endoscopic management. The most common site of involvement was duodenum (5/10) followed by both pylorus and duodenum (4/10) and pylorus (1/10). Most of the strictures were short web-like, and the mean (SD) number of stricture was 2.0 (0.94). Endoscopic balloon dilatation was successful in 90% (9/10) cases requiring mean (SD) of 2.0 (1.6) sessions of dilatation to achieve target diameter of 15 mm and mean (SD) of 5.3 (2.7) sessions to maintain it over a treatment period of 4.5 months (IQR 2–15 months). There was no procedure-related complication or mortality.

## 1. Introduction

Peptic ulcer disease and corrosive ingestion are the leading causes of benign gastric outlet obstruction [1]. Nonsteroidal anti-inflammatory drugs (NSAIDs) are known to be associated with various forms of gastrointestinal injuries including peptic ulcer disease, diaphragm disease of the bowel, and protein losing enteropathy [2]. NSAIDs are one of the most commonly prescribed medications, and they are often used for long period of time. Chronic NSAID consumption is a rare cause of gastric outlet obstruction [3–5]. There are case reports of duodenal web-like strictures associated with long-term NSAID use [6].

Gastric outlet obstruction (GOO) includes obstruction in the antropyloric area or in the bulbar or postbulbar duodenal segments [1]. With the advent of EBD, endoscopic therapy has become the cornerstone of management of benign gastric outlet obstruction. A number of reports have reported the safety and efficacy of the procedure [7, 8]. The initial experience of EBD was under fluoroscopic guidance; subsequently it has been shown that EBD can be performed under endoscopic guidance only [9]. We here report our experience on gastric outlet obstruction caused by NSAIDs.

## 2. Methods

Between January 2004 and December 2010, all consecutive patients with symptomatic NSAID-induced GOO were evaluated. Details of NSAID intake were noted in terms of type, amount, and duration of intake. All patients underwent upper gastrointestinal endoscopy, barium meal follow through, and contrast-enhanced computed tomography to note the site and length of gastric and duodenal stricture(s) and to rule out presence of jejunal and ileal involvement. Patients who satisfied the selection criteria were subjected to endoscopic balloon dilatation by using through-the-scope (TTS) balloon dilators. Inclusion criteria were (1) symptomatic GOO with postprandial vomiting and (2) narrowing of the pyloroantral area or duodenum on gastroduodenoscopy and barium meal follow through examination. Patients with (1) >2.5 cm narrowing in the pyloroantral area and (2) inability to give informed consent were excluded. Informed written consent was obtained from each patient at each session, and the study was approved by the institute ethics committee.

Dilatation was carried out by using wire-guided TTS CRE (controlled radial expansion) balloon (Boston Scientific Corp, Marlborough, Mass, USA), after premedication with intravenous midazolam (Fulsed; Ranbaxy, Mumbai, India) and n-hyoscine butyl bromide (Buscopan; Cadila healthcare, Goa, India). The barium examination films of each patient were kept in view during dilatation. The diameter of the balloon was selected on the basis of the endoscopist's subjective assessment of the severity of the stenosis. The balloon was negotiated across the narrowed segment under endoscopic vision and was positioned approximately equally on either side of the narrowing. Inflation was done by using a saline-solution-filled syringe mounted on a pressure gun (Alliance inflation device; Boston Scientific Corp, Marlborough, Mass, USA) as per manufacturer's instructions. The CRE balloon was inflated to incremental diameters, for 60 seconds at each diameter. After deflation, the area was observed for signs of bleeding, and an attempt was made to negotiate the scope through the narrowed segment. In patients with multiple strictures, the procedure was repeated at each narrowing. Patients were observed for 4 hours after the procedure for pain in the abdomen, tachycardia and a drop in blood pressure, and only clear liquids were allowed for 24 hours. Dilatation was repeated at 2-3-week intervals until a 15 mm diameter balloon could be passed through the stricture. All patients were given proton pump inhibitors in daily doses until the end of dilatation. Once dilatation to 15 mm was achieved, patients were followed up every 2 weeks and were questioned about their symptoms, and endoscopy was performed. During followup endoscopic dilatation was performed till there was no residue in two consecutive endoscopies. Successful dilatation was defined by the achievement of target diameter (15 mm), absence of symptoms, and no residue in two consecutive endoscopies. After successful dilatation, all patients were followed at 2-month intervals until December 2010. Recurrence was defined by the reappearance of symptoms and failure to pass the diagnostic scope in the previously negotiable stricture.

## 3. Statistical Analysis

Parametric quantitative variables were expressed as mean (SD); nonparametric quantitative variables were expressed as median with interquartile range (IQR). Categorical variables were expressed as percentages. Statistical analysis was performed using the statistical software package SPSS version 17.0 (SPSS, Chicago, Illinois, USA).

## 4. Results

Baseline characteristics of the cases are described in Table 1. Mean (SD) age of the cases was 45.2 (16.9) years; there were 8 males and 2 females. Median duration of symptoms was 9 months (IQR 1–120 months). All the patients had presented with vomiting (100%), one (12.5%) patient had history of abdominal pain, and 3 (37.5%) patients had weight loss. All the patients had history of consuming NSAIDs. The number of tablets taken per day ranged from 1 to 8; the dose of each NSAID taken per day was as per the available strength in the market multiplied by the average number of tablets consumed per day. The median duration of NSAID consumption was 8 years (IQR 3–20 years) (Table 1). 

**Figure 1:**
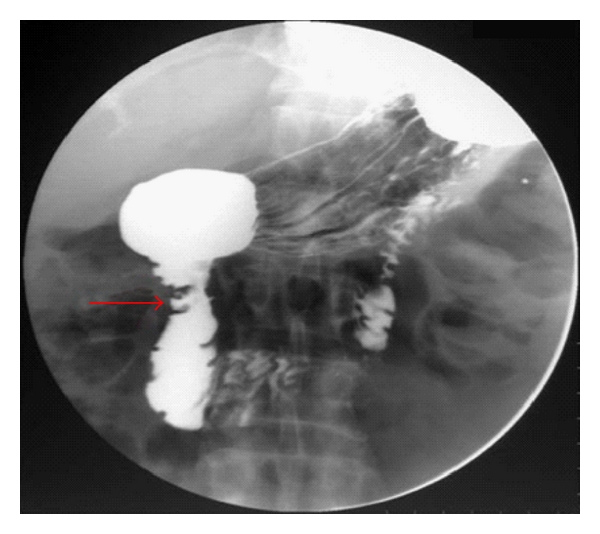
Barium meal examination showing two small web like strictures at the junction of the 1st and 2nd parts of duodenum with proximal dilatation (arrow).

**Figure 2:**
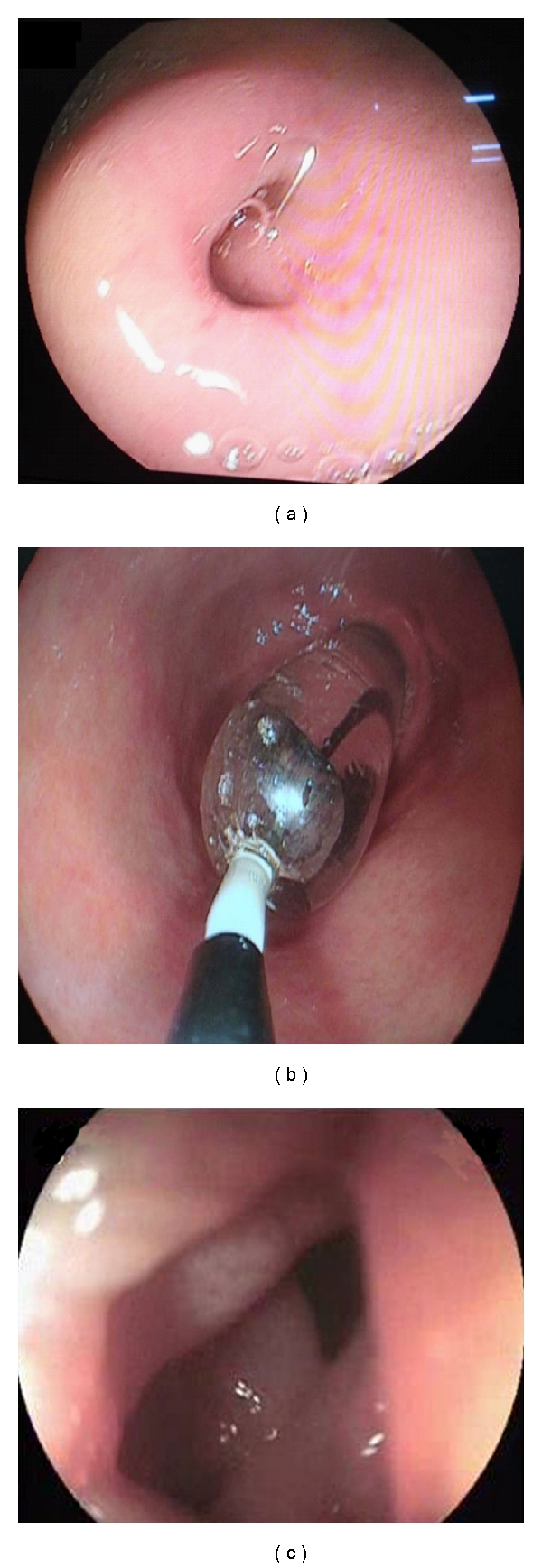
Upper gastrointestinal endoscopy showing circular stricture at the junction of the 1st and 2nd parts of duodenum (a), controlled radial expansion balloon in situ (b), and after dilatation the scope was negotiable into the 2nd part of duodenum (c).

**Figure 3:**
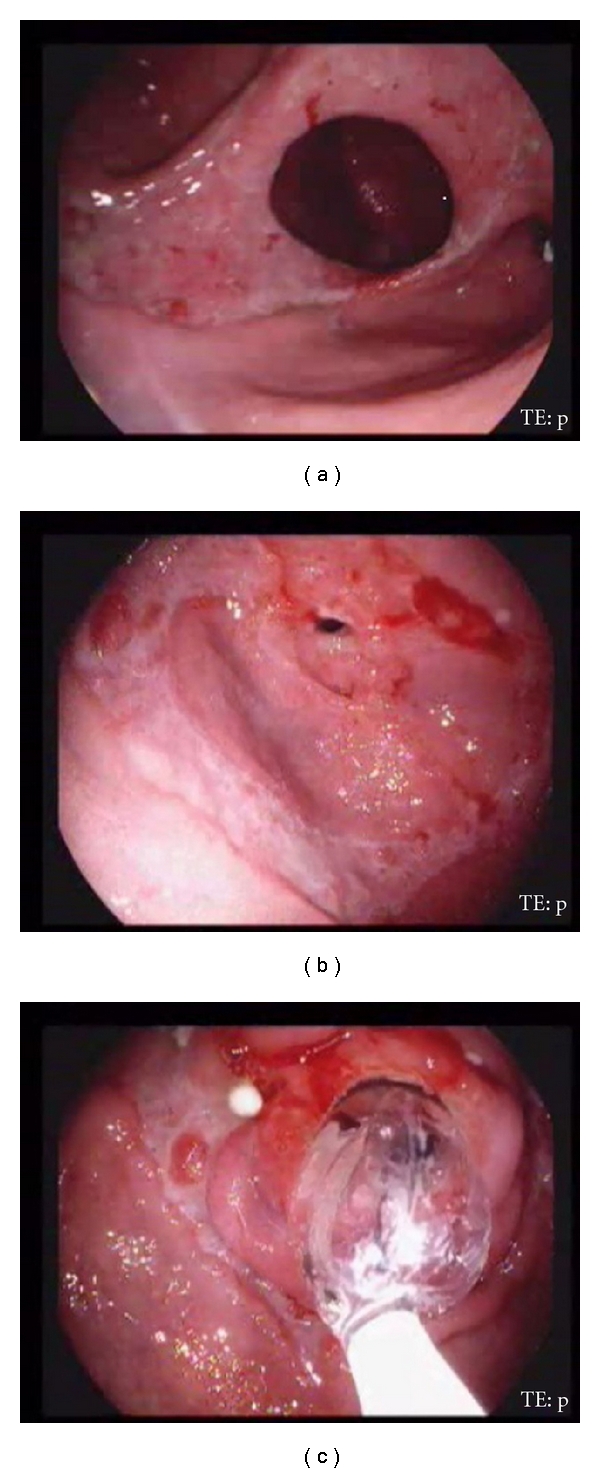
Upper gastrointestinal endoscopy of another patient showing pyloric stricture with large antral ulcer (a) and another stricture at the junction of the 1st and 2nd parts of duodenum (b), and strictures were dilated with controlled radial expansion balloon (c).

The site of involvement of stricture was pylorus in 5 (50%) patients, duodenum in 9 (90%) patients, and in 4 (40%) patients both the sites were involved, only pylorus was involved in one patient only. The strictures were in the form of short 2-3 mm web-like circumferential narrowing, except in 2 patients with pyloric stenosis who had longer (~5 mm) segments of narrowing (Figure 1). The mean (SD) number of strictures was 2.0 (0.94). Ulceration at the rim of stricture was noted in 3 (30%) patients. All the patients underwent endoscopic stricture dilatation using CRE balloon (Figures 2, 3). The mean (SD) number of dilatations to achieve target diameter of 15 mm was 2.0 (1.6). The mean (SD) number of dilatations required to maintain was 5.3 (2.7). Median weight gain was 5 kgs (IQR 2–14 kgs), and the median duration of treatment was 4.5 months (IQR 2–15 months). These patients were followed up to a median of 12 months (IQR 2–16 months). There was no recurrence during this period. There were no complications like bleeding or perforation, and there was no mortality. Nine (90%) patients were managed successfully; only one patient (case no. 2) required surgery because of failure of endoscopic therapy. He had two web-like strictures in the second and third parts of duodenum. Though his strictures could be dilated to 15 mm in 3 sessions, yet he presented with persistent symptoms and his gastric residue did not decrease. He was consuming nimesulide for 20 years. He underwent surgery in the form of gastrojejunostomy. 

## 5. Discussion

In this study we have described the role of endoscopic dilatation in patients of NSAID-induced pyloroduodenal strictures leading to GOO. Nine of the 10 patients could be successfully treated using EBD, with no recurrence. There were no procedure-related complications. Only one patient required surgical management.

Malignancy remains the commonest cause of GOO but benign etiologies like peptic ulcer disease and caustic ingestion are responsible for significant proportion of such patients [10]. NSAID ingestion is an uncommon cause of GOO [3, 4]. Mechanism of GOO caused by NSAID abuse is not entirely clear. Diminished levels of prostaglandin E2 have been implicated in the pathogenesis of gastric outflow obstruction by causing pyloric edema and scarring [11]. Increased histamine release leads to increased gastric secretion, reduction of mucosal absorption, and gastric motility disturbances [11]. This suggests a possible mechanism whereby NSAIDs might predispose to gastric outflow obstruction.

NSAIDs can cause strictures anywhere in the gastrointestinal tract right from the esophagus to the colon [2, 12, 13]. Small bowel strictures caused by NSAIDs are short (2-3 mm) web-like and are often labeled as diaphragms; they have been mostly reported in jejunum and ileum [2]. The predilection for duodenum in our patients may be due to more frequent use of nonenteric-coated preparations in India.

EBD is now an established treatment modality in the management of benign GOO. Response to endoscopic dilatation depends upon the etiology, length, and site of stricture [4]. Patients with chronic pancreatitis-related duodenal stricture have been reported to have the worst outcome with endoscopic dilatation [4]. Need for more than two sessions of endoscopic dilatation to relieve symptom is also a predictor for failure of endoscopic therapy in patients with peptic ulcer disease [14]. 

There is scanty data on the use of EBD in patients with NSAID-induced gastric outlet obstruction. In a study from India, out of 3 patients with NSAID-induced duodenal webs, two could be managed successfully with endoscopic therapy in the form of radial incision of webs with mixed cutting and coagulation current using a sphincterotome [6]. Apart from this there are only case reports of endoscopic management of NSAID-induced GOO [15]. The present study is the largest series on successful management of NSAID-induced pyloroduodenal obstruction using EBD. With the advent of double balloon enteroscopy NSAID-induced jejunal and ileal strictures have also been reported to respond to endoscopic dilatation [16]. 

The target diameter for EBD for duodenal strictures is not defined. Whereas esophageal strictures need to be dilated to >14 mm and dysphagia occurs with diameter <13 mm, pyloric dilatation is targeted at 15 mm [8, 17, 18]. The duodenum has much larger diameter than pyloric canal, and there are no guidelines on the optimal diameter of dilatation. We could achieve a diameter of 15 mm in mean of 2 sessions of EBD, but nearly all the patients had persistence of some narrowing at follow-up endoscopy along with persistent gastric residue. Keeping in view our experience with EBD in caustic-induced GOO, we continued with dilatation with a 15 mm balloon till there was no residue in 2 consecutive endoscopies and there was no symptom. At this stage all the patients had easily negotiable strictures. There was no recurrence over a median follow-up period of 12 months, although it was short in the last 3 patients.

The main advantage of using EBD for NSAID-induced GOO is avoidance of surgery in 90% of patients. The drawback is that the patient has to come for repeated dilatation; this requires long-term commitment and compliance, median treatment period being 4.5 months. On the other hand surgery remains a one-time procedure, but it has significant morbidity. 

To conclude, NSAID-induced pyloroduodenal strictures are an uncommon cause of GOO and can be managed successfully in a majority of patients with EBD. Surgery can be reserved only for failure of EBD.

## Figures and Tables

**Table 1:** Baseline characteristics of patients and results of endoscopic dilatation.

Case	Age/gender	Symptoms	NSAIDs, tablet strength	Number of tablets consumed per day	Duration of NSAIDs intake (years)	Site of involvement	Number of strictures	Total number of dilatation	Duration of treatment (months)	Follow up (months)	Outcome
1	50/M	Vomiting	Aspirin 325 mg	1	3	D1-D2, D2, D3	3	7	4	12	Successful
2	35/M	Vomiting	Nimesulide 100 mg	3	20	D2, D3	2	3	15	15	Unsuccessful, required surgery
3	40/M	Vomiting	Nimesulide 100 mg, Aspirin 325 mg	5	9	Pylorus	1	4	5	15	Successful
4	19/M	Vomiting	Diclofenac 50 mg	6	3	Pylorus, D1-D2, D2-D3	3	6	6	16	Successful
5	51/F	Vomiting, weight loss	Ibuprofen 400 mg	6	4	D1-D2	1	10	9	9	Successful
6	40/M	Vomiting	Nimesulide 100 mg	4	12	Pylorus, D2-D3, D3-D4	3	9	12	12	Successful
7	85/M	Vomiting	Ibuprofen 400 mg	2	20	D1-D2	1	2	2	12	Successful
8	40/M	Vomiting, pain abdomen, weight loss	Diclofenac 50 mg	8	8	Pylorus, D1-D2, D2-D3	3	5	3	3	Successful
9	40/M	Vomiting	Ibuprofen 400 mg, Diclofenac 50 mg	4	12	Pylorus, D1-D2	2	5	2	2	Successful
10	52/M	Vomiting, weight loss	Ibuprofen 400 mg	6	7	D1-D2	1	2	3	2	Successful

D1: first part of duodenum, D2: second part of duodenum, D3: third part of duodenum, NSAIDs: nonsteroidal anti-inflammatory drugs.

## Abbreviations

NSAIDs: Nonsteroidal anti-inflammatory drugs
GOO: Gastric outlet obstruction
EBD: Endoscopic balloon dilatation.
